# Supplementary figures and images for: Correlates of disease severity in bluetongue as a model of acute arbovirus infection
Source: PLoS Pathog. 2024 Aug 16;20(8):e1012466. doi: 10.1371/journal.ppat.1012466 (PMC11357116; doi:10.1371/journal.ppat.1012466)

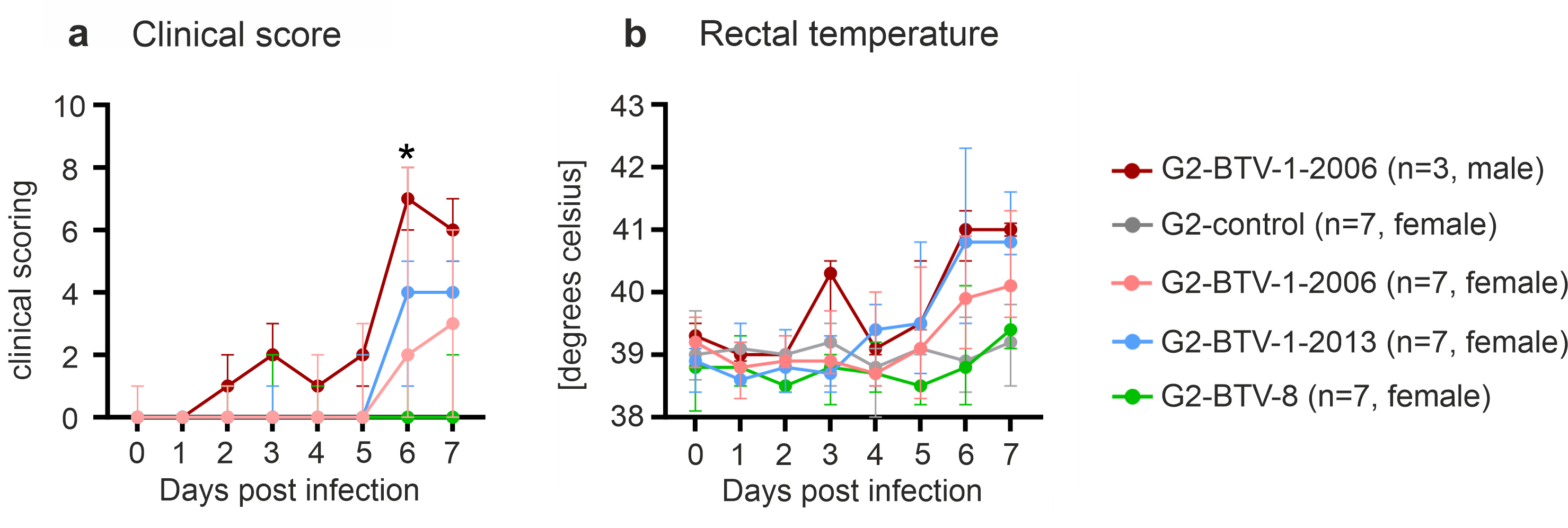

Supplement: S1 Fig — Three additional rams were experimentally infected in location G2 and compared to female sheep. (a) Clinical signs. Note significant difference in the severity of clinical signs in infected rams compared to ewes at 6 dpi (p = 0.0344; 2-way-ANOVA). (b) Rectal temperature. Only male animals infected with BTV-1 2006 have a fever peak at 3 dpi compared to all other groups; no significant differences between male and female animals infected with BTV-1 2006 have been detected at any time point. Data are shown as median, minimum and maximum values. (TIF) [file ppat.1012466.s001.tif]

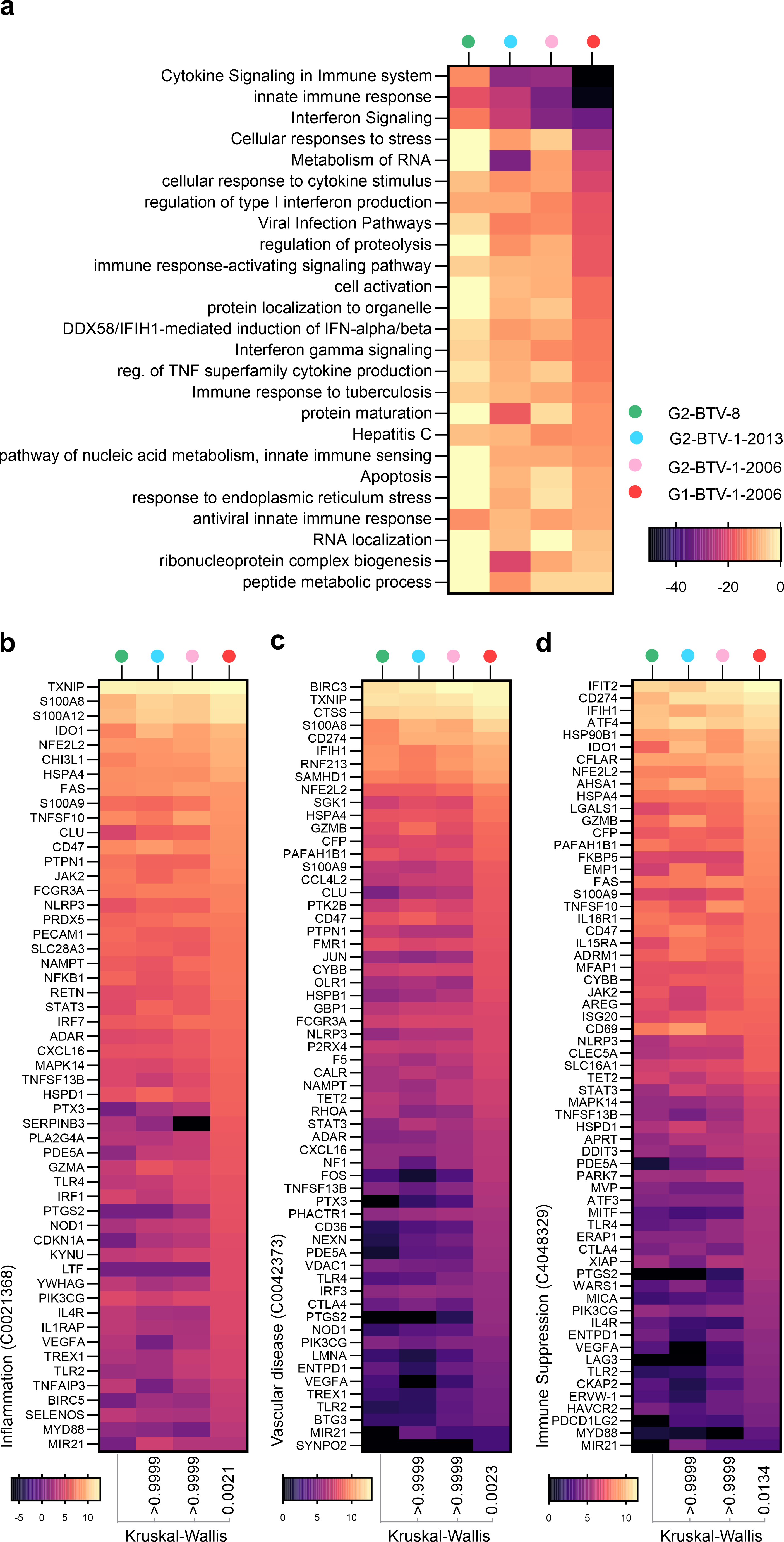

Supplement: S2 Fig — RNA was extracted from BTV infected sheep at 7 days post infection (dpi) and used for RNA-Seq. Host reads were aligned to the sheep genome and normalised to their corresponding negative control group (counts per million; CPM). Differentially expressed genes (adjusted p-value < 0.05; FDR) were identified for each pairwise comparison (as indicated). (a) Metascape pathway analysis of up-regulated DEGs showing relative pathway enrichment for each pairwise comparison (p-value < 0.05; log10 p-values shown). Top 25 pathways shown (ranked on G1-BTV-1-2006). (b-d) Expression profile (mean log2 CPM) of host DEGs identified during BTV infection associated with DisGeNET inflammation (C0021368), vascular disease (C0042373), and immune suppression (C4048329) pathways. Missing values are plotted as zero. P-values and test shown. (TIF) [file ppat.1012466.s002.tif]

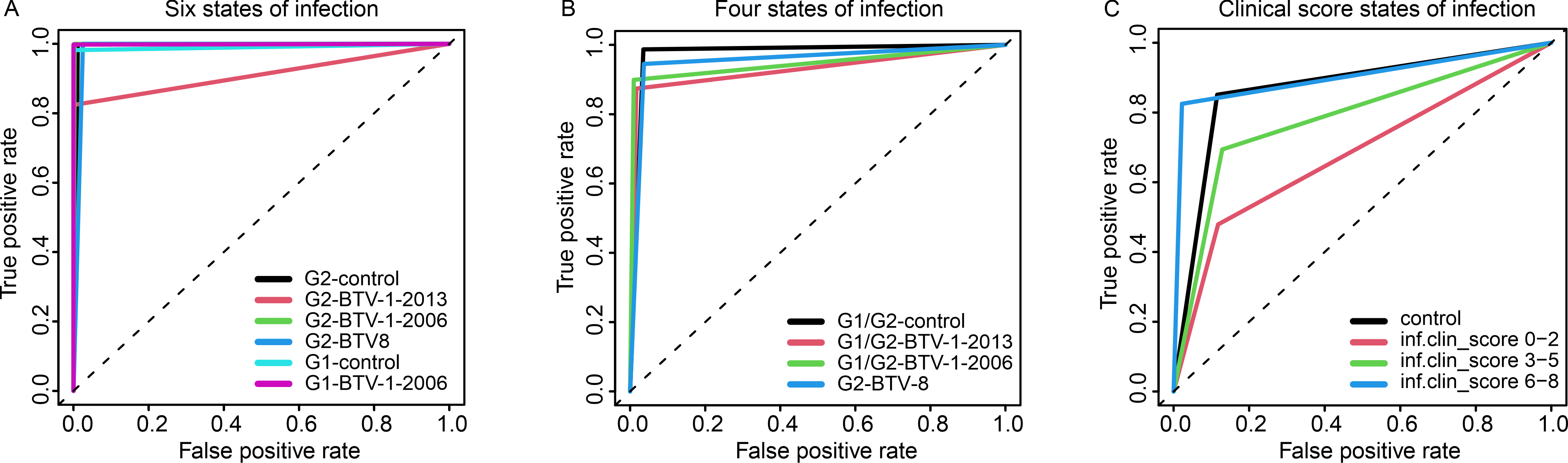

Supplement: S3 Fig — A) True and false positive rate is shown for six states of infection (a), for four states of infection (b) and clinical states (c). (TIF) [file ppat.1012466.s003.tif]
