# Supplementary material for: Correlates of disease severity in bluetongue as a model of acute arbovirus infection
Source: PLoS Pathog. 2024 Aug 16;20(8):e1012466. doi: 10.1371/journal.ppat.1012466 (PMC11357116; doi:10.1371/journal.ppat.1012466)
Supplement: S1 Table — (PDF) [file ppat.1012466.s004.pdf]

Table S1. Animals and study design.

Main Studies

	Groups		
**G1**	G1-BTV-1-2006	G1-BTV-1-2013	G1-control
Length of study scheduled	21 dpi	21 dpi	21 dpi
Postmortem	7-9 dpi	21 dpi	21 dpi
Animals per group (n)	7	7	7
Sex/breed	Male/Sarde	Male/Sarde	Male/Sarde
Location (city, country)	Teramo, Italy	Teramo, Italy	Teramo, Italy

	Groups			
**G2**	G2-BTV-1-2006	G2-BTV-1-2013	G2-BTV-8	G2-control
Length of study scheduled	7 dpi	7 dpi	7 dpi	7 dpi
Postmortem	7 dpi	7 dpi	7 dpi	7 dpi
Animals per group (n)	7	7	7	7
Sex/breed	Female/Sarde	Female/Sarde	Female/Sarde	Female/Sarde
Location (city, country)	Sassari, Italy	Sassari, Italy	Sassari, Italy	Sassari, Italy

Additional groups

	Virus		
	BTV-1_{IT2006}	BTV-1_{IT2006}	Mock
Length of study scheduled	2 dpi	7 dpi	2 dpi
postmortem	2 dpi	7 dpi	2 dpi
Animals per group (n)	4	3	3
Sex/breed	Female/Sarde	Female/Sarde	Female/Sarde
Location (city, country)	Sassari, Italy	Sassari, Italy	Sassari, Italy
