# Supplementary material for: Correlates of disease severity in bluetongue as a model of acute arbovirus infection
Source: PLoS Pathog. 2024 Aug 16;20(8):e1012466. doi: 10.1371/journal.ppat.1012466 (PMC11357116; doi:10.1371/journal.ppat.1012466)
Supplement: Attachment — Submitted filename: response letter_mpv3_final.docx [file ppat.1012466.s018.docx]

Xiu-Feng Wan
Guangxiang Luo
Michael Malim

PLoS Pathogens

02 July 2024

Dear Editors

RE: Point-by-point response letter, PPATHOGENS-D-24-00497 “Correlates of Disease Severity in Acute Arbovirus Infection” by Herder et al.

Thank you for editing our manuscript. We would also like to thank the reviewers for the overall positive and constructive reviews. We have clarified the points raised by Reviewer # 1. In particular, we have run an additional machine learning programme (Boruta) to reinforce the data and added ROC curves.

To facilitate the editorial process, we cut and pasted each of the editorial comments within this letter (in red) and addressed them individually. In addition, we have provided a version of the revised manuscript with track changes compared to the originally submitted manuscript.

REVIEWER 1

*Major: 1. Line 178 ‘We used recursive feature elimination to find the most predictive core subset of parameters distinguishing each group’. A quantitative measurement, i.e., slope or something, or a kind of grid search, needs to be adopted to increase the scientific rigor of the studies. From the naked eye, from Figure 2, it is arbitrary to set up these thresholds to select the top parameters of the model. The same issue with many ‘arbitrary’ numbers in the study, such as Line 182, ‘…required a 182 minimum of 50…’, why 50?*

We thank the reviewer for highlighting this and understand the comment. We would like first to stress that we used machine learning only as an initial screen to highlight those parameters among the 300+ for which we had data that were worthy of being subsequently investigated and validated experimentally. The conclusions of the study come from the experimental validation of those parameters highlighted experimentally.

We agree that the initial number of parameters selected for further classification was done arbitrarily at the point the curves plateaued. We aimed to select the point where prediction impact of each additional parameter was likely from stochastic noise, rather than by using a different quantitative cut off method. The goal of the machine learning approach was simply to “filter” the parameters collected (n=332) for those that had the potential to be more informative. But the actual conclusions of the study comes only from those parameters that have been subsequently validated experimentally.

The models built from the chosen parameters were robust and had predictive value as shown in Table 1 (for the 6 states of infection) and Table 2 (for 4 states of infection) and the confusion matrices. In addition, we followed the reviewer’s suggestions and run Boruta as an additional analysis – confirming with this approach identified by random forest and clarified the confusion matrices in addition to add ROC curves (see below).

To make sure that we highlight the point identified by the reviewer we highlighted how the choice of some of these parameters was arbitrary as follows:

Abstract: page 2, lines 38: *“We identified and experimentally validated five different fundamental processes affecting the severity of bluetongue: ..”*

Page 8, line 203 (version ‘tracked changes’), “*We selected arbitrarily the number of parameters based on where the slope of the prediction accuracy curve plateaued, indicative of redundancy of additional parameters (Fig. 2 a-c; Tables 1 to 3).”*

*Major 2. Line 187: ‘We used 1000 rounds of cross-validation, each using randomly selected 5 animals from each class to train the model and 2 unseen animals’. Cross validation is usually used to check the model’s performance, the proportion of these 1000 does not necessarily reveal the feature and data’s performance and importance. Instead, a confusion matrix, ROC curve, and precision-recall curve can be used to quantify the performance and robustness.*

We agree with the reviewer, as indeed the output of the cross validation is a confusion matrix which we had added in the manuscript, but we didn’t highlight it sufficiently. We produced two sets of confusion matrices for each model, one with the real data, and another with randomised data points (in the manuscript called “Shuffled data”). These were originally submitted in the manuscript as Tables S6, S7 and S8 for the shuffled data and tables 1, 2 and 3 for the confusion matrices for the real unshuffled models. From these matrices we just presented the proportions as a convenient summary in the main text. We think the vast discrepancy of the performance of the models on the real and shuffled data provides very strong evidence of the models’ performance.

In the revised manuscript, we also added the ROC curves (plotting the type I and type II error trade-offs in our imperfect predictive signal) as we agree with the reviewer that they provide a useful visualisation of these tables. We generated these curves and added them a supplementary data (Figure S3).

We have modified the text to make it clearer that we have included the confusion matrices and added the ROC curves as supplementary Figure S3.

Page 10, lines 246-248 (version ‘tracked changes’) “*In addition, to further confirm our ML results were robust and not due to overfitting on background noise, we trained random forests on shuffled data tables (confusion matrices), where each sample's data was randomly re-assigned, removing the link between infection state, and collected data*.”

Page 10, lines 251-252 (version ‘tracked changes’): *“ROC (receiving operating characteristics) curves, plotting the type I and type II error trade-offs in our imperfect predictive signals, are shown in Figure S3.”*

*It may be good to have some other algorithms other than Random Forest to check the robustness of the feature selected. For example, Boruta (https://cran.r-project.org/web/packages/Boruta/Boruta.pdf) may be a good candidate.*

We thank the reviewer for the suggestion of the Boruta package which we included in the revised version, although we also reiterate that the robustness of the parameters identified has been validated experimentally, as opposed to mathematically.

We have now investigated our random forest parameter importance by using the Boruta algorithm (Kursa and Rudnicki 2010) and included this approach in the text and in Tables S10-S12.

In particular in the Results section, page 10 lines 238-245 (version ‘tracked changes’): “*We also run the Boruta algorithm (Kursa and Rudnicki, 2010) for 5000 iterations to confirm the random forest approach: 17 out of 17 parameters from the four state list are in the 60 parameter confirmed list from Boruta (Table S6); 50/50 parameters from the six state parameter list are in the 93 parameter confirmed list from Boruta (only 1 parameter was tentative for this model) (Table S7); less parameters (39/100) from the clinical score list are in the confirmed list of 64 parameters from Boruta (Table S8). These low values reflect also the low predictive values of the parameters identified by random forest as the “clinical states” are defined by the clinical scores of the infected animals, which in turn are based mostly on subjective values of the observers.*”

We also added Boruta in the Methods; page 25, lines 646-655 (version ‘tracked changes’): *The confusion matrices showing the lack of prediction power of these models can be found in Tables S6, S7 and S8, while the ROC curves are shown in Figure S3. In addition, we used the Boruta algorithm to confirm the data obtained by machine learning (Kursa and Rudnicki, 2010). This algorithm creates a “shadow parameter” column for each parameter in the model, shuffling the values randomly across samples. The importance metrics are then calculated for the real and shadow parameters, and a parameter is deemed as “confirmed” if it shows higher importance values than these shuffled shadow parameters. Parameters are determined as “unimportant” if they produce values significantly lower than the maximum importance of the shadow parameters. If a parameter cannot be determined as confirmed or unimportant in the initial number of runs, it is marked as “tentative”.*

*Reviewer #1: 1. Line 264: Importantly, analysis of blood transcriptome using standard pathway analysis methods also revealed “cytokine signaling in immune system” (GO:0019221), “innate immune responses” (GO:0002226) and “interferon signalling” (GO:0060337). It could be better to have p-value or FDR here to demonstrate the significance of GO terms.*

We thank the reviewer to point this out as the requested data set shows better the significance of GO terms. Therefore, we uploaded two files as supplemental data as these tables contain the individual gene lists and enrichment information used to identify the pathways.

File Table S13 is a copy of the log10 P-values used for Fig. S2A. This gives the p-values for each cell shown in the heatmap.

Pathway values can be found in the file “Annotation” with the label Table S14. LogP and log(q-value) can be found in here under the “enrichment tab”.

We added the Tables in the text on page 12, lines 300-301: *... upregulated pathways discriminating the different clinical outcomes of bluetongue infection (Fig. S2; Tables S****13****, and S****14****).*

*2. Typo in Short Title: ‘…in Arboviral dDsease’*

We correct the typo in the revised version.

Reviewer #2:

*Herder and colleagues described correlates of disease severity in acute bluetongue virus (BTV) infection in sheep. Authors used three bluetongue viruses showing different clinical signs to infect sheep and then employed machine learning to identified determinants of virus and host associated with disease pathogenesis. They identified 5 factors critical for clinical disease and pathogenesis. Then authors conclude that using an agnostic machine learning approach is a good tool to understand pathogenetic mechanisms affecting the disease outcome of an arbovirus infection. This manuscript is well-written and this study providing some interesting results*.

We thank the reviewer #2 for the positive evaluation.

*Reviewer #2: 1) The title looks like not fit very well as only BTV was tested in one species, not other arboviruses. Not sure whether the same results will be obtained in other viruses in different species.*

We have modified the title as suggested into: “Correlates of Disease Severity in Bluetongue as a Model of Acute Arbovirus Infection”

*Reviewer #2: 2) Based on the results, the sex is critical for clinical disease, especially the male sheep show more severe disease than the female. Although author tested 332 biological parameters, likely no clear explanations on it and suggest discussing it.*

Yes, we agree with the reviewer that it does appear that ram are more susceptible to severe disease than females, although to fully determine this point one would have to design the experiment exactly for this goal and likely using large experimental groups (which are difficult to achieve in a larger animal CL3 facility.

We Have added this point in the Discussion:

Page 17, lines 440-443 (version ‘tracked changes’): “*The data we obtained also suggest that rams display a worse clinical outcome to BTV infection than ewes, however further experimental studies will be required to conclusively address this point. Usually, in domestic flocks there are several dozes ewes for each ram, hence it is difficult to extrapolate solid data with regards to sex-bias from the naturally occurring outbreaks of bluetongue.”*

*Reviewer #2: 3) The study design did not consider the animal age. Normally BTVs can cause more severe disease in young animals. Should discuss it what will be turned out if young animals used.*

The reviewer is correct in that we used adult sheep or similar age throughout the experiment. Considering these studies need to be carried out in large animal CL3 facilities, it would be beyond the scope of the study to also assess different ages. I would, however, point out that there are no solid data conclusively pointing out to age differences in the clinical outcome of bluetongue.

There is a very old study making this point: (J. Spreull, Malarial catarrhal fever (bluetongue) of sheep in South Africa. Journal of Comparative Pathology and Therapeutics, 18 (1905), pp. 321-337). However, in one of our own experimental studies, comparing adults with 8-month-old lambs, we did not observe significant differences. (Caporale M, Di Gialleonorado L, Janowicz A, Wilkie G, Shaw A, Savini G, Van Rijn PA, Mertens P, Di Ventura M, Palmarini M. Virus and host factors affecting the clinical outcome of bluetongue virus infection. J Virol. 2014 Sep;88(18):10399-411. doi: 10.1128/JVI.01641-14. Epub 2014 Jul 2. PMID: 24991012; PMCID: PMC4178883.

We re-arranged the numbering of the Supplementary Tables based on the new data, which have been added. Furthermore, we highlighted the typos we corrected in the revised version.

We thank you again for editing our manuscript and we hope that is now ready for publication in PLOS Pathogens.

Your Sincerely,

**Massimo Palmarini OBE FMedSci FRSE**

Director and Chair of Virology

MRC-University of Glasgow Centre for Virus Research

T: +44 (0)141 330 2356

E: [massimo.palmarini@glasgow.ac.uk](mailto:massimo.palmarini@glasgow.ac.uk)
